# Absence of Cold-Inducible RNA-Binding Protein (CIRP) Promotes Angiogenesis and Regeneration of Ischemic Tissue by Inducing M2-Like Macrophage Polarization

**DOI:** 10.3390/biomedicines9040395

**Published:** 2021-04-07

**Authors:** Matthias Kübler, Sebastian Beck, Silvia Fischer, Philipp Götz, Konda Kumaraswami, Hellen Ishikawa-Ankerhold, Manuel Lasch, Elisabeth Deindl

**Affiliations:** 1 Walter-Brendel-Centre of Experimental Medicine, University Hospital, Ludwig-Maximilians-Universität München, 81377 Munich, Germany; Matthias.Kuebler@med.uni-muenchen.de (M.K.); sebastian.beck@med.uni-muenchen.de (S.B.); P.Goetz@med.uni-muenchen.de (P.G.); Kumaraswami.Konda@med.uni-muenchen.de (K.K.); Hellen.Ishikawa-Ankerhold@med.uni-muenchen.de (H.I.-A.); manuel_lasch@gmx.de (M.L.); 2 Biomedical Center, Institute of Cardiovascular Physiology and Pathophysiology, Faculty of Medicine, Ludwig-Maximilians-Universität München, 82152 Planegg-Martinsried, Germany; 3 Department of Biochemistry, Faculty of Medicine, Justus Liebig University, 35392 Giessen, Germany; Silvia.Fischer@biochemie.med.uni-giessen.de; 4 Department of Internal Medicine I, Faculty of Medicine, University Hospital, Ludwig-Maximilians-Universität München, 81377 Munich, Germany; 5 Department of Otorhinolaryngology, Head and Neck Surgery, University Hospital, Ludwig-Maximilians-Universität München, 81377 Munich, Germany

**Keywords:** angiogenesis, cold-inducible RNA-binding protein, CIRP, CIRBP, neutrophil extracellular traps, NETs, macrophage polarization, inflammation, tissue regeneration, ischemia

## Abstract

Cold-inducible RNA-binding protein (CIRP) is an intracellular RNA-chaperone and extracellular promoter of inflammation, which is increasingly expressed and released under conditions of hypoxia and cold stress. The functional relevance of CIRP for angiogenesis and regeneration of ischemic muscle tissue has never been investigated and is the topic of the present study. We investigated the role of CIRP employing CIRP deficient mice along with a hindlimb model of ischemia-induced angiogenesis. 1 and 7 days after femoral artery ligation or sham operation, gastrocnemius muscles of CIRP-deficient and wildtype mice were isolated and processed for (immuno-) histological analyses. CIRP deficient mice showed decreased ischemic tissue damage as evidenced by Hematoxylin and Eosin staining, whereas angiogenesis was enhanced as demonstrated by increased capillary/muscle fiber ratio and number of proliferating endothelial (CD31^+^/BrdU^+^) cells on day 7 after surgery. Moreover, CIRP deficiency resulted in a reduction of total leukocyte count (CD45^+^), neutrophils (myeloperoxidase, MPO^+^), neutrophil extracellular traps (NETs) (MPO^+^/CitH3^+^), and inflammatory M1-like polarized macrophages (CD68^+^/MRC1^-^), whereas the number of tissue regenerating M2-like polarized macrophages (CD68^+^/MRC1^-^) was increased in ischemic tissue samples. In summary, we show that the absence of CIRP ameliorates angiogenesis and regeneration of ischemic muscle tissue, most likely by influencing macrophage polarization in direction to regenerative M2-like macrophages.

## 1. Introduction

The dysregulation of new blood vessel formation in the adult individual plays a significant role in many acute and chronic diseases [1,2]. Insufficient angiogenesis leads to impaired wound healing in damaged tissue, whereas uncontrolled hyperstimulation can promote growth and nutrition supply in cancer and further facilitates its blood vessel-dependent metastatic spread [3,4,5]. Clinical therapy is necessary for both promoting and inhibiting angiogenesis in different kinds of diseases. The recent approval of anti-vascular endothelial growth factor (VEGF) antibodies as a treatment against age-related macular degeneration and various cancer types highlights the success in this research field [6,7]. On the other hand, there are still many hurdles to overcome in other angiogenesis-related diseases, such as rheumatoid arthritis, wound healing, and ischemic diseases [8,9,10]. Angiogenesis in particular plays an important role in regeneration of ischemic tissue after myocardial infarction, peripheral arterial disease, and stroke, as it has a direct impact on the disease-associated long-term effects and mortality [11,12,13]. Consequently, the investigation of the endogenous regulators affecting the growth and diminution of adult microcirculation plays a significant role in developing a wide range of therapies.

Arteries increase their supplying capacities through circumferential enlargement and remodeling processes. Natural bypass formation from pre-existing vasculature in response to increased shear stress is called arteriogenesis [14,15,16,17]. In contrast, capillary networks grow in complexity and increase their total surface by a modification referred to as angiogenesis [18].

Angiogenesis describes the process of forming new capillaries from pre-existing vasculature, thereby increasing the capillarity of tissue under different pathological and physiological conditions [18]. There are two mechanisms of angiogenesis: (a) by splitting vessels of a pre-existing capillary network, which is declared as intussusceptive angiogenesis, or (b) by sprouting angiogenesis, a mechanism defined as the formation of new capillary branches by migration and proliferation of endothelial cells, creating new capillary beds [19,20,21]. Hypoxia in ischemic tissue has been described as the major angiogenic trigger for both forms of angiogenesis, whereas shear stress has been discussed only to induce intussusceptive angiogenesis [19]. Angiogenesis occurs during embryonic development as well as in the adult individual [22]. It plays a fundamental role in the female menstrual cycle, pregnancy, wound healing, skeletal and cardiac muscle hypertrophy as well as in ischemic tissue regeneration [3,4,5,18,23,24,25,26].

The process of angiogenesis depends on the balance of various pro and anti-angiogenic signals. The involvement of vascular components, such as endothelial cells and pericytes, and their interaction with their surroundings, comprising the extracellular matrix, immune cells, growth factors, and inhibitors, regulate the pro- or anti-angiogenic growth of the microvasculature. It can result in endothelial cell migration and proliferation, extracellular proteolysis, endothelial cell differentiation, and stabilization [18,22].

The most crucial, actually known pro-angiogenic regulator is the vascular endothelial growth factor A (VEGFA) [18,27,28]. VEGFA belongs to a family of powerful pro-angiogenic factors, including placental growth factor (PIGF), VEGFB, VEGFC, and VEGFD [29]. In a dose-dependent manner, the binding of VEGFA to the receptor tyrosine kinase VEGF receptor 2 (VEGFR-2) can promote endothelial cell differentiation, proliferation, and angiogenic remodeling, whereas hyperstimulation can even lead to angioma formation [30]. It has been described that potent suppliers of VEGFA are myeloid cells, such as neutrophils and monocytes, thus playing an important role in influencing angiogenesis not only by clearing cell debris but also by controlling VEGFA bioavailability [18,31,32,33,34,35,36].

The discovery of neutrophil extracellular traps (NETs) presented a significant advance in leukocyte immunology [37]. NETs are neutrophil-originated chromatin filaments with citrullinated histone H3 (CitH3) accompanied by enzymes such as myeloperoxidase (MPO), which get released upon various inflammatory stimuli [38,39]. A link between NETs and their effect on angiogenesis was described in 2016, stating that NETs can promote angiogenesis in vitro and in vivo. NETs increase intercellular adhesion molecule 1 (ICAM-1) expression and influence endothelial cell migration and progenitor cell recruitment through VEGF-signaling [40]. Further, it was described that NETs support tissue remodeling in retinopathy via degradation of senescent vasculature [41]. Yet, excessive accumulation of NETs at the side of inflammation has been associated with prolonged tissue repair and aggravated cell damage [42,43]. Showing cytotoxic effects on endothelial cells in a dose-dependent manner, histones of NETs are thought to be the source of this detrimental cell harm [44]. Therefore, a sensitive balance of NET formation is believed to improve angiogenesis without impairing tissue restoration.

Angiogenesis can also be regulated by RNA binding proteins that regulate RNA dynamics, such as subcellular localization and metabolism [45,46,47]. The cold-inducible RNA-binding protein (CIRP) belongs to the glycine-rich RNA binding protein family, with an RNA recognition motif (RRM) and a carboxyl-terminal domain containing several arginine/glycine-rich (RGG) motifs [48]. The RRM of CIRP can regulate a great variety of cellular processes via binding of the target RNAs 3′ untranslated region (UTR), influencing RNA processing, translation, and turnover [49,50,51]. CIRP is expressed in various tissues and cell types. The activation and translocation from the nucleus to the cytoplasm are mediated in response to cellular stress, for instance, oxidative stress, hypoxia, and mild hypothermia [48,50,51,52,53,54]. Through the binding of RNA targets, intracellular CIRP (iCIRP) is thought to help the cell to deal with such stresses. The targets include transcripts responsible for a redox protective effect, telomerase elongation, DNA repair, mechanisms against apoptosis, and hypoxia [51,54,55,56,57,58]. This response to hypoxia is particularly interesting since the expression of VEGF is induced by hypoxia too [59]. Thus, CIRP is involved in many diseases, e.g., neurodegenerative, cardiovascular, many types of cancers, and genetic and developmental diseases [60].

Recently, its role as a secreted extracellular protein acting as a damage-associated molecular pattern (DAMP) was discovered [49,50,51,54,61,62,63,64,65]. The devastating effects of extracellular CIRP (eCIRP) were firstly described in hemorrhagic shock [66]. The hypoxia-dependent liberation to the extracellular space leads to tissue damage and aggravated inflammation. Because the transcription of CIRP is enhanced due to cold stress and hypoxia, it leads to translocation and release into the extracellular space [66]. Since both conditions are found in ischemic tissue, we hypothesized that CIRP, intra- and extracellularly, might be involved in ischemia-induced angiogenesis and the concomitant tissue repair after arterial occlusion.

Furthermore, it was discovered that eCIRP promotes the formation of a newly found subset of pro-inflammatory neutrophils in sepsis. Those neutrophils were characterized by increased expression levels of C-X-X chemokine receptor type 4 (CXCR4), ICAM-1, inducible nitric oxide synthase (iNOS), reactive oxygen species (ROS), and NETs [67]. It was also shown that eCIRP excessively induces NET formation by upregulating the expression of peptidylarginine deiminase 4 (PAD4), which is essential for catalyzing the citrullination of histones, and by the triggering receptor expressed on myeloid cells 1 (TREM-1) dependent Rho-GTPase activation [38,68,69,70].

A study on arteriogenesis and microRNAs discovered the presence of CIRP in cells of the arteriolar wall. It has been suggested that iCIRP could possibly affect the processes of arteriogenesis and angiogenesis through the regulation of posttranscriptional processing of specific microRNAs belonging to the 14q32 locus. By blocking a known target from this cluster, miR-329, the perfusion recovery improved after induced arteriogenesis and angiogenesis by arterial obstruction [71,72,73]. It has been shown that iCIRP can bind to miR-329 in vitro, leading to the hypothesis that iCIRP may affect arteriogenesis and angiogenesis via the regulation of microRNAs.

Another recent study showed that the absence of CIRP improves wound healing of skin lesions. An accelerated inflammatory state mirrored by a reduced number of GR1^+^ leukocytes and an increased number of CD31^+^ cells were found in the CIRP deficient mice, proposing a faster tissue regeneration and improved angiogenesis in the absence of CIRP [74].

Here, we investigated the role of CIRP in sterile and ischemia-induced angiogenesis and tissue regeneration in association with leukocyte infiltration and macrophage polarization.

## 2. Materials and Methods

### 2.1. Animals and Treatments

All experimental interventions were approved by the Bavarian Animal Care and Use Committee (ethical approval code: ROB-55.2Vet-2532.Vet_02-17-99, approved at the 8 December 2017) and were performed in strict accordance with the German animal legislation guidelines. For the investigations of the mRNA levels of CIRP, C57BL/6J (Charles River Laboratories, Sulzfeld, Germany) mice, aged 8-12 weeks, were sacrificed (n = 3) per analyzed time point. For all the other experiments, adult male CIRP-knockout C57BL/6NCrSlc or sibling wildtype mice (n = 5), aged 8–12 weeks, were used. Mice were housed in a temperature-controlled room on a 12 h light/dark circle and fed a standard laboratory diet. To evaluate cell proliferation after 7 days of femoral artery ligation, mice were injected with 100 µl BrdU (bromodeoxyuridine) (Sigma-Aldrich, St. Louis, MO, USA) (12.5 mg/mL BrdU in phosphate-buffered saline (PBS, PAN Biotech, Aidenbach, Germany, pH 7.4)) i.p. daily, starting immediately after the surgical procedure.

### 2.2. Femoral Artery Ligation and Tissue Processing

To initiate angiogenesis in the gastrocnemius muscle of the lower hindlimb, the right hindlimb’s femoral artery was unilaterally ligated, while the left side was sham-operated and served as an internal control, as previously described [75]. Before surgery, the mice were initially anesthetized with a combination of fentanyl (0.05 mg/kg, CuraMED Pharma, Karlsruhe, Germany), midazolam (5.0 mg/kg, Ratiopharm GmbH, Ulm, Germany), and medetomidine (0.5 mg/kg, Pfister Pharma, Berlin, Germany). For tissue collection 1 or 7 days after FAL, mice were again anesthetized as described above, and the hindlimbs were perfused with adenosine buffer (1% adenosine (Sigma-Aldrich), 5% bovine serum albumin (BSA, Sigma-Aldrich), dissolved in PBS), followed by 3% paraformaldehyde perfusion (PFA, Merck, Darmstadt, Germany) (for cryopreservation) or 4% PFA (Merck) (for paraffin embedding) in PBS, pH 7.4. After perfusion was completed, both gastrocnemius muscles of each mouse were extracted and stored appropriately. For immunohistology, tissue was embedded in Tissue-Tek compound (Sakura Finetek Germany GmbH, Staufen, Germany) and cryopreserved at −80 °C. For paraffin histological analyses, tissue was embedded in paraffin and stored at room temperature (RT) until further processing.

### 2.3. Histology and Immunohistology

The cryoblocks of the gastrocnemius muscle from day 1 and 7 after FAL were cut in 10 µm thick slices and processed for immunohistology. The samples for BrdU-staining were treated with 1 N HCl for 30 min at 37 °C, before permeabilization with 0.2% Triton X-100 solution (AppliChem GmbH, Darmstadt, Germany) in 1 × PBS/0.1% Tween-20 (AppliChem GmbH)/0.5% BSA for 2 min. Subsequently, blocking solution (10% goat serum (Abcam, ab7481, Cambridge, UK) in 1 × PBS/0.1% Tween-20/0.5% BSA) was applied for 30 min and the primary anti-BrdU-antibody (Abcam, ab6326, dilution 1:50 in 10% goat serum) was incubated at 4 °C overnight. Secondary staining was performed with a goat anti-rat Alexa Fluor®-546 antibody (Invitrogen, Thermo Fischer Scientific, A-11081, Carlsbad, CA, USA, dilution 1:100) for 1 h at RT. After the second blocking with 1 × PBS/0.1% Tween-20/4% BSA for 30 min at RT, the tissue was incubated with an anti-CD31-Alexa Fluor® 647 antibody (Biolegend, 102516, San Diego, CA, USA, dilution 1:50 in 1 × PBS/0.1% Tween-20) used as endothelial cell marker, together with an anti-ACTA2-Alexa Fluor® 488 antibody (actin alpha 2) (Sigma-Aldrich, F3777, dilution 1:400 in 1 × PBS/0.1% Tween-20) used as pericytes marker for 2 h at RT. For total leukocyte staining, anti-CD45-Alexa Fluor® 488 antibody (BioLegend, 11-0451-85, dilution 1:100 in 1 × PBS/0.1% Tween-20) was applied at 4 °C overnight. Macrophages were labeled with an anti-CD68-Alexa Fluor® 488 antibody (Abcam, ab201844, dilution 1:200 in PBS) at 4 °C overnight and co-incubated with an anti-MRC1 (mannose receptor C-type 1) antibody (Abcam, ab64693, dilution 1:200 in PBS) as M2-like macrophage polarization marker, at 4 °C overnight, followed by a secondary antibody staining with a donkey-anti-rabbit Alexa Fluor® 546 (Invitrogen, A-10040) for 1 h at RT. To stain for NETs, incubation with the primary antibodies anti-myeloperoxidase (MPO; R&D Systems, AF3667, Minneapolis, MN, USA, dilution 1:20 in 10% donkey serum (Abcam, ab7475) in 1 × PBS/0.1% Tween-20/0.5% BSA) and anti-citrullinated histone H3 antibody (Cit-H3; polyclonal rabbit anti-Histone H3 (citrulline R2+R8+R17), Abcam, ab5103, dilution 1:100 in 10% donkey serum in 1 × PBS/0.1% Tween-20/0.5% BSA) was performed at 4 °C overnight. Secondary antibody staining was done with a donkey anti-goat Alexa Fluor® 594 (Invitrogen, A-11058, dilution 1:100 in 1 × PBS/0.1% Tween-20) and a donkey anti-rabbit Alexa Fluor® 488 antibody (Invitrogen, A-21206, dilution 1:200 in 1 × PBS/0.1% Tween-20) for 1 h at RT. All tissues were incubated with DAPI (Thermo Fisher Scientific, 62248, dilution 1:1000 in PBS) for 10 min at RT to label nucleic DNA. For sample mounting, an antifade mounting medium (Dako, Agilent, Santa Clara, CA, USA) was used. Gastrocnemius muscle sections from ischemic (occluded) and non-ischemic (sham-operated) side collected at day 7 were stained for different leukocyte populations (pan-leukocytes and macrophages) and capillaries, whereas muscle tissue from day 1 was only used for neutrophil and NETs staining. We used a confocal laser scanning microscope LSM 880 (Carl-Zeiss Jena GmbH, Jena, Germany) with a 20× objective (415 µm × 415 µm) as well as an epifluorescence microscope (Leica DM6 B, Leica microsystems, Wetzlar, Germany) with a 20× objective (630 µm × 475µm) to investigate 5 defined fields of view for each muscle to count cells, muscle fibers and NETs. CD31/ACTA2/BrdU/DAPI and CD45/DAPI staining were analyzed with the epifluorescence microscope, whereas CD68/MRC1/DAPI and MPO/CitH3/DAPI stains were studied with the confocal laser scanning microscope. To assess the process of angiogenesis, we calculated the capillary (CD31^+^ACTA^-^ cells were considered as endothelial cells) per muscle fiber ratio as previously described [76].

Hematoxylin and Eosin staining was performed on 5 µm thick paraffin sections, according to the manufacturer’s instruction (Carl Roth GmbH, Karlsruhe, Germany), from the gastrocnemius muscles of CIRP-wildtype and CIRP-knockout mice (n = 5) collected on day 7 after surgical intervention. The damaged area of the total gastrocnemius muscle area was imaged with an epifluorescence microscope (Leica DM6 B) and analyzed with AxioVision Rel. 4.8. software (Carl-Zeiss Jena GmbH). NETs 3D rendering was performed with Imaris (Version 9.2, Oxford Instruments, Abingdon, UK) software.

### 2.4. Cell Culture

The monocyte/macrophage mouse cell line J774A.1 was obtained from Merck and cultured in DMEM medium (Gibco, Thermo Fisher Scientific) containing 2 mM glutamine (Gibco) and 10% fetal calf serum (FCS, Gibco). Before stimulation, cells were washed once with PBS and incubated for 2 h in a serum-free cell culture medium containing different concentrations of recombinant murine CIRP (Hölzel Diagnostika, Cologne, Germany).

### 2.5. Quantitative Real-Time PCR (qRT-PCR)

Following treatment of J774A.1 with different concentrations of CIRP as indicated in the legends of the corresponding figure, cells were washed twice with PBS, lysed, and RNA was isolated with the total RNA extraction kit (Peqlab, VWR, Radnor, PA, USA). For qRT-PCR analyses, 1 µg of RNA was reverse-transcribed using the High-Capacity cDNA Reverse Transcription Kit (Applied Biosystems, Carlsbad, CA, USA) and DNA amplification was performed with a StepOne Plus cycler (Applied Biosystems) in a reaction volume of 10 µl using the SensiMix Sybr Kit (Bioline, Luckenwalde, Germany) with 50 pmol of each primer. To avoid amplification of genomic DNA, primers were designed to span exon-exon junctions. The qPCR was performed under the following conditions: an initial denaturation step at 95 °C for 8.5 min followed by 45 cycles, consisting of denaturation (95 °C, 30 s), annealing (60 °C, 30 s) and elongation (72 °C, 30 s). Melt curve analysis was performed to control specific amplification. Results were normalized to the expression levels (*E*) of actin and expressed as the ratio of *E*(target)/*E*(Actin). The following mouse primers were used: TNF-α forward TGG TTT GTG AGT GTG AGG GTC, TNF-α reverse ACT GAA CTT CGG GGT GAT CG, IL-6 forward CTC TGC AAG AGA CTT CCA TCC A, IL-6 reverse TTG TGA AGT AGG GAA GGC CG, Arg2 forward CCC TCC CTG CCA ATC ATG T, Arg2 reverse CTA GCT TCT TCT GTC CCC GAG, YM1 forward CTG TGG AGA AAG ACA TTC CAA, YM1 reverse AAG AGA CTG AGA CAG TTC AGG, MRC1 forward CAA GCA GCA GAA TGC TGA CC, MRC1 reverse AGT CCA ATC CAG AGT CCC GA, actin forward CGCGAGCACAGCTTCTTTG, and actin reverse CGTCATCCATGGCGAACTGG.

The gastrocnemius muscles were isolated from C57BL/6J mice without femoral artery ligation (basal), or 12 h, 24 h, and 72 h after femoral artery ligation or sham operation, homogenized and RNA was extracted using MaXtract tubes (QIAGEN GmbH, Hilden, Germany) and phenol-chloroform (Merck). Genomic DNA was removed using an RNase-free DNase kit (Promega GmbH, Walldorf, Germany). 1 µg of total RNA was used for reverse transcription to cDNA (Maxima™ H Minus cDNA-Synthese Master Mix, Thermo Fisher Scientific). qPCR was performed as described above using PowerUp™ SYBR® Green Mastermix (Thermo Fisher Scientific), with the annealing temperatures for CIRP primer at 62 °C, and for 18S rRNA primer at 64 °C. The expression level of CIRP was normalized to the expression level of the 18S-rRNA and data were calculated as described above. The following primers were used: CIRP forward AGG GTT CTC CAG AGG AGG AG, CIRP reverse CCG GCT GGC ATA GTA GTC TC, 18S-rRNA forward GGA CAG GAT TGA CAG ATT GAT AG, 18S-rRNA reverse CTC GTT CGT TAT CGG AAT TAA C (Eurofins Genomics, Ebersberg, Germany).

### 2.6. Statistical Analyses

GraphPad Prism 8 (GraphPad Software, La Jolla, CA, USA) was used for the statistical analyses and graphic plotting. Data are means ± standard error of the mean (S.E.M.). Statistical analyses were indicated in the figure legends and considered statistically significant at *p* < 0.05.

## 3. Results

To investigate the role of CIRP in angiogenesis and repair in ischemic muscle tissue, we used a well-established murine hindlimb model described by Limbourg et al. [75]. In this model, femoral artery ligation (FAL) results in arteriogenesis in the adductor muscle in the upper leg and, due to reduced perfusion, in ischemic tissue damage and angiogenesis in the gastrocnemius muscle of the lower leg.

**Figure 1:**
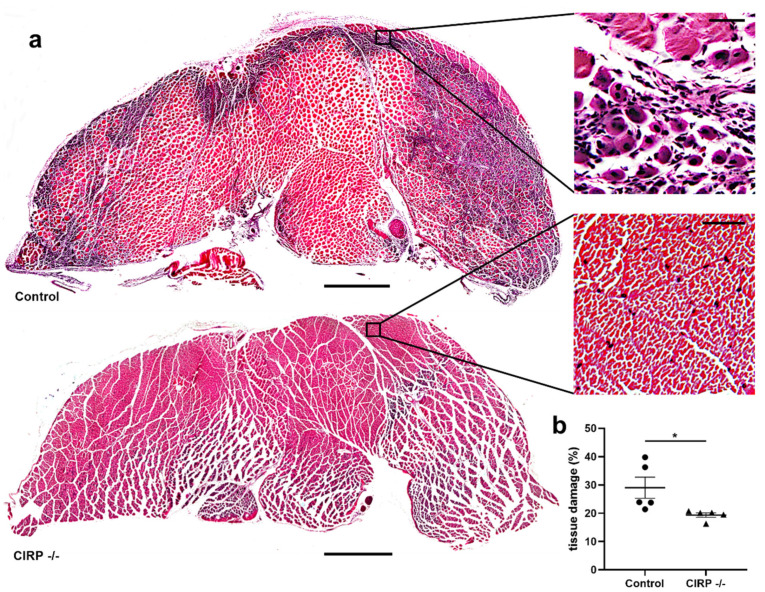
Cold-inducible RNA-binding protein (CIRP) knockout mitigates ischemic tissue damage. (**a**) Representative pictures of Hematoxylin and Eosin stained gastrocnemius muscle slices of control (above) and CIRP -/- (below) mice 7 days after femoral artery ligation (FAL). Right: Magnification of the areas indicated by the black square. Regenerating muscle cells showing centralized nuclei are a sign of ischemic muscle damage. Scale bars 1000 µm (overview) and 50 µm (detail). (**b**) The scatter plot displays the percentage of tissue damage in relation to the whole gastrocnemius muscle of control and CIRP -/- mice 7 days after FAL. The total gastrocnemius cross-sectional area (about 20 mm^2^) was analyzed. Data are means ± S.E.M., n = 5 per group, * *p* < 0.05 (CIRP -/- vs. control) by unpaired, two-sided student’s t-test.

To analyze the extent of tissue damage in gastrocnemius muscles, we performed Hematoxylin and Eosin staining of the ischemic (occluded) and non-ischemic (sham-operated) tissue samples isolated 7 days after the surgical intervention. As expected, we did not find any tissue damage in the sham-operated muscles, either in CIRP deficient (CIRP -/-) nor in wildtype control mice (data not shown). FAL resulted in ischemic tissue damage in gastrocnemius muscles of all mice, however, it was significantly reduced in CIRP -/- mice compared to wildtype mice (Figure 1a,b). Results of qRT-PCR showed significantly increased expression levels of CIRP mRNA in gastrocnemius muscles of CIRP wildtype mice 72 h after the arterial occlusion (Figure S1).

**Figure 2:**
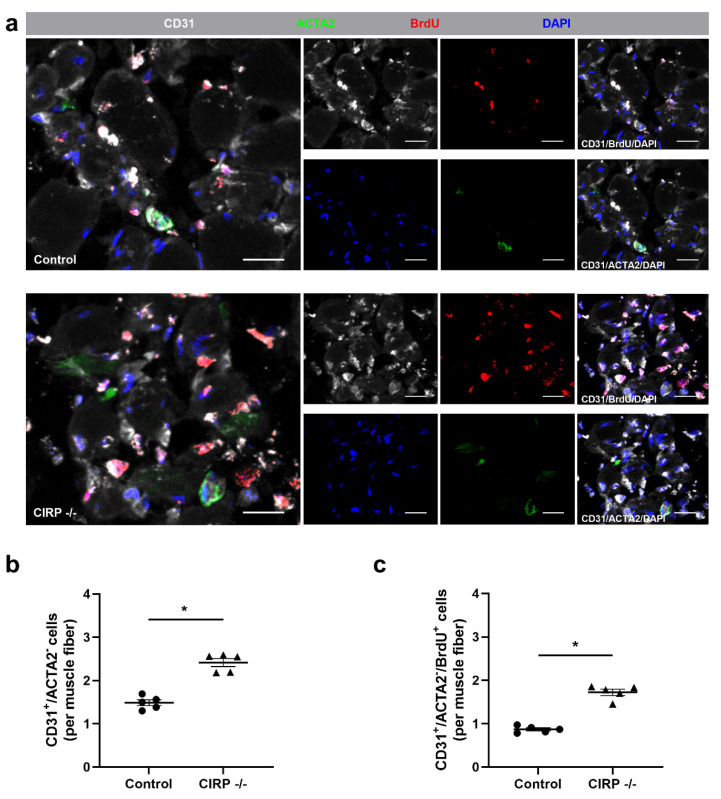
Cold-inducible RNA-binding protein (CIRP) knockout enhances capillary growth. (**a**) Representative immunofluorescence stains of ischemic gastrocnemius muscle slices of CIRP-wildtype control (above) and -knockout (below) mice 7 days after femoral artery ligation (FAL). Smaller images of single- and merged (CD31/BrdU/DAPI and CD31/ACTA2/DAPI) channels, large images with all merged channels of endothelial cells (anti-CD31, white), proliferating cells (anti-BrdU (bromodeoxyuridine), red), pericytes (anti-ACTA2 (actin alpha 2), green), and nucleic acid (DAPI, blue). Scale bars 20 µm. (**b**) The scatter plots display CD31^+^ACTA2^−^ (endothelial) cells and (**c**) CD31^+^ACTA2^−^BrdU^+^ (proliferating endothelial) cells per muscle fiber of ischemic gastrocnemius muscles of CIRP -/- and control mice 7 days after FAL. Data are means ± S.E.M., n = 5 per group. * *p* < 0.05 (CIRP -/- vs. control) by unpaired, two-sided student’s *t*-test.

To evaluate the effect of CIRP deficiency on angiogenesis, we performed CD31/ACTA2/BrdU/DAPI quadruple immunofluorescence staining (Figure 2a) and calculated the capillary to muscle fiber ratio in the areas of ischemic damage in gastrocnemius muscle tissue, again isolated 7 days after (FAL). CD31 was used as an endothelial cell marker, actin alpha 2 (ACTA2) as a vascular mural cell marker that stains pericytes in tissue microvasculature [77,78,79,80]. Hence, CD31^+^ACTA2^-^ cells were defined as capillaries. 

Compared to control mice, CIRP -/- mice showed a significant increase in the capillary to muscle fiber ratio, which was used as an indicator for angiogenesis (Figure 2b). We also analyzed the capillary to muscle fiber ratio, including only proliferating (BrdU^+^, bromodeoxyuridine) endothelial cells. Again, the values were significantly increased in CIRP -/- mice compared to wildtype control mice (Figure 2c). Without induction of ischemia (sham operation), there was no significant difference in the capillary to muscle fiber ratio between CIRP -/- and wildtype control mice (Figure S2).

**Figure 3:**
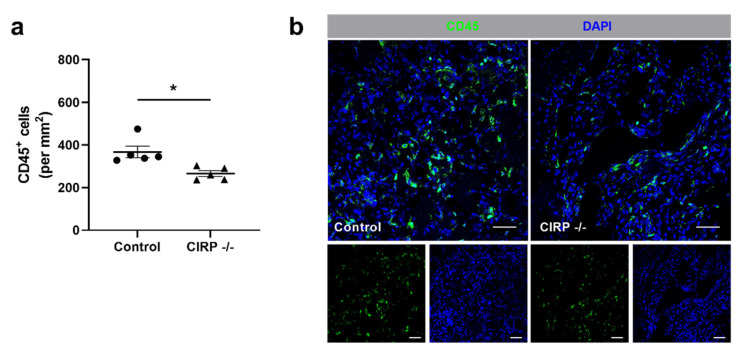
Cold-inducible RNA-binding protein (CIRP) knockout decreases leukocyte infiltration in ischemic tissue. (**a**) The scatter plot displays the relative amount of CD45+ (pan-leukocyte marker) cells (per mm^2^) of ischemic gastrocnemius muscles of wildtype and CIRP -/- mice 7 days after femoral artery ligation (FAL). Data are means ± S.E.M., n = 5 per group. * *p* < 0.05 (CIRP -/- vs. control) by unpaired, two-sided student’s t-test. (**b**) Representative immunofluorescence stainings of ischemic gastrocnemius muscles of CIRP wildtype control (left) and CIRP -/- (right) mice 7 days after FAL. Smaller images display single channels, large images merged channels of leukocytes (anti-CD45, green) and nucleic acid (DAPI, blue) stains. Scale bars 50 µm.

Besides promoting vascular cell proliferation, leukocytes play a major role in removing cell debris and tissue repair. Therefore, we examined the effect of CIRP deficiency on leukocyte recruitment by immunofluorescence staining of tissue labeled with an anti-CD45 antibody as a pan-leukocyte marker. On day 7 after the surgical intervention, the number of CD45^+^ cells was significantly lower in damaged tissue of CIRP -/- mice compared to CIRP-wildtype mice (Figure 3a,b). In tissue samples of non-ischemic (sham-operated) legs, no significant differences were found in the number of CD45^+^ cells in control vs. CIRP -/- mice (data not shown).

**Figure 4:**
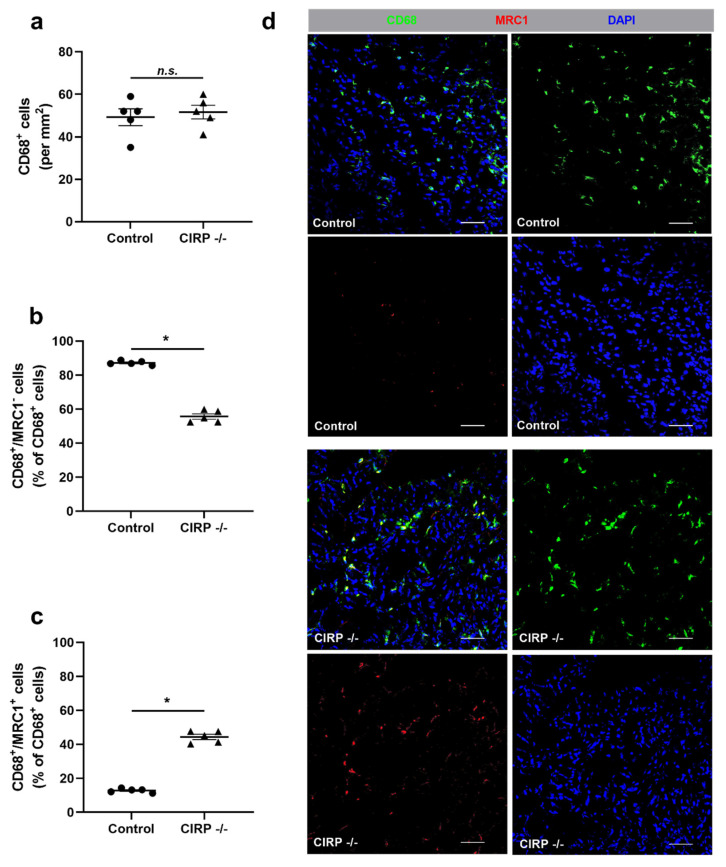
Cold-inducible RNA-binding protein (CIRP) knockout affects macrophage polarization. (**a**) The scatter plots display the relative amount of CD68^+^ cells (macrophages) (per mm^2^), (**b**) CD68^+^/MRC1^-^ (mannose receptor c-type 1) (M1-like polarized macrophages), and (**c**) CD68^+^/MRC1^+^ cells (M2-like polarized macrophages) in relation to all CD68^+^ cells (in%) in ischemic gastrocnemius muscles of CIRP wildtype control and CIRP -/- mice 7 days after femoral artery ligation (FAL). Data are means ± S.E.M., n = 5 per group. n.s. *p* > 0.05 ((**a**) CIRP -/- vs. control), * *p* < 0.05 ((**b**,**c**) CIRP -/- vs. control) by unpaired, two-sided student’s t-test. (**d**) Representative immunofluorescence staining of ischemic gastrocnemius muscle slices of wildtype control (above) and CIRP -/- mice (below) 7 days after FAL. Images show single and merged channels of CD68 and MRC1 labeled macrophages (anti-CD68, green; anti-MRC1, red) and nucleic acid (DAPI, blue). Scale bars 50 µm.

To further investigate the different leukocyte subpopulations, we performed a macrophage staining using CD68 as a macrophage marker and mannose receptor C-type 1 (MRC1) as a marker for M2-like polarization. The total number of CD68^+^ (macrophage) cells recruited to the ischemic area of gastrocnemius muscles did not significantly differ between the CIRP -/- and wildtype control mice (Figure 4a). However, our results demonstrated that the number of M1-like polarized macrophages (CD68^+^MRC1^-^) was significantly lower, whereas the number of M2-like polarized macrophages (CD68^+^MRC1^+^) was significantly higher in ischemic gastrocnemius muscles of CIRP deficient mice in comparison to wildtype mice at day 7 after FAL (Figure 4b–d). Tissue samples isolated from the contralateral non-ischemic (sham-operated) sides showed no significant differences in the number of CD68^+^ cells as well as their polarizations (data not shown). Furthermore, we investigated the effect of recombinant CIRP on macrophages in vitro. Here, we found that the application of recombinant CIRP on J774A.1 macrophages induced the expression of the M1-like polarization markers tumor necrosis factor alpha (TNF-α), interleukin 6 (IL-6), and arginase 2 (Arg2), but showed no effect on the expression of the M2-like polarization markers MRC1 and chitinase-like protein 3 (Ym1) (Figure S3).

**Figure 5:**
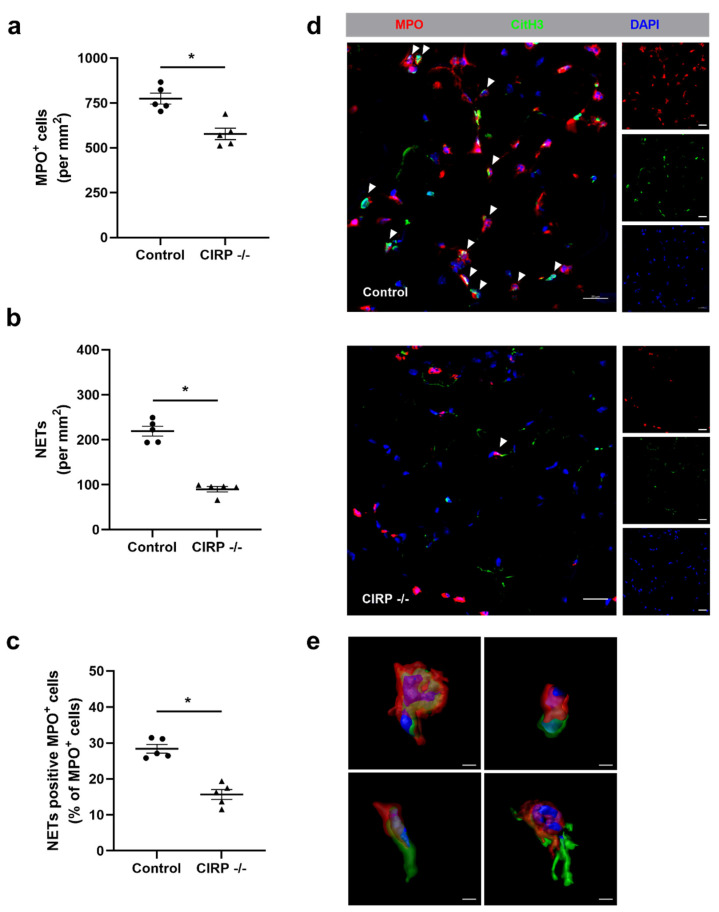
Absence of Cold-inducible RNA-binding protein (CIRP) interferes with neutrophil recruitment and neutrophil extracellular trap (NET) formation in ischemic muscle tissue. The scatter plots display the number of (**a**) MPO^+^ (myeloperoxidase, marker for neutrophils) cells, (**b**) NETs (both per mm^2^), and (**c**) NET positive MPO^+^ cells per total MPO^+^ cells in ischemic gastrocnemius muscles isolated from control and -/- mice 1 day after femoral artery ligation (FAL). Data are means ± S.E.M., n = 5 per group. * *p* < 0.05 (CIRP -/- vs. control) by unpaired, two-sided student’s t-test. (**d**) Representative immunofluorescence staining of ischemic gastrocnemius muscle slices of CIRP wildtype control (above) and CIRP -/- (below) mice 1 day after FAL. Images display single and merged channels of neutrophils (MPO^+^) and NETs (MPO^+^/CitH3^+^) (citrullinated histone 3) (indicated by white arrowheads) labeled with anti-MPO (red), anti-CitH3 (green), and DAPI (nucleic acid, blue). Scale bars 20 µm. (**e**) Representative immunofluorescence image of detailed 3D rendering images showing NET formation in different stages. Scale bars 3 µm.

An anti-MPO stain was used to identify neutrophils in gastrocnemius muscles isolated 1 day after the surgical procedure. Compared to wildtype control mice, the number of neutrophils was significantly lower in ischemic tissue samples of CIRP deficient mice (Figure 5a,d). To quantify the number of neutrophils in the process of NET formation, we used an anti-CitH3, an anti-MPO, and a DAPI labeling. Here, our data demonstrated that the number of NETs was significantly reduced in the CIRP -/- compared to wildtype control mice (Figure 5b,d,e).

Additionally, we calculated the percentage of NETs related to the total neutrophils count to investigate whether the absence of CIRP shows an effect on the NET formation of neutrophils in ischemic tissue. Again, the percentage of NET positive neutrophils was significantly reduced in CIRP -/- mice compared to wildtype control mice (Figure 5c,d). The non-ischemic (sham-operated) gastrocnemius muscles of CIRP -/- and wildtype control mice did not show any difference regarding neutrophil accumulation, NET formation, and the number of neutrophils forming NETs (data not shown).

## 4. Discussion

In the present study, we analyzed the impact of CIRP deficiency on ischemia-induced angiogenesis and tissue repair along with the accumulation of leukocytes in a murine hindlimb model. Our results indicate that the complete absence of CIRP ameliorates angiogenesis as well as tissue regeneration in murine ischemic muscle tissue, as shown by an increased capillary to muscle fiber ratio and reduced tissue damage. This effect may be partly attributed to the predominance of the regenerative anti-inflammatory M2-like polarized macrophages, as well as to the reduced numbers of neutrophils and NETs in the ischemic tissue of CIRP deficient mice.

In the murine hindlimb model of ischemia, ligation of the femoral artery results in impaired blood flow to the lower limb, consequently resulting in ischemia and local tissue damage and fibrosis [75]. While trying to preserve metabolism in the ischemic area and clearing the accumulated cell debris, the processes of angiogenesis in association with local leukocyte recruitment get induced [18]. In the used model, ischemic tissue damage in the gastrocnemius muscle is also dependent on collateral artery growth efficiency in the adductor muscle of the upper leg. Collateral growth gets induced after the femoral artery is ligated and the blood flow is redirected in its deep branch. Whether the decreased tissue damage in CIRP deficient mice results from ameliorated angiogenesis alone or also improved arteriogenesis in the upper leg still has to be analyzed. Increased mRNA levels of CIRP after FAL in the adductor muscle have been reported for a severe murine hindlimb ischemia model [72]. In the present study, we found increased mRNA levels of CIRP in the gastrocnemius muscle 72 h after induction of angiogenesis. From previous in vitro investigations on CIRPs binding to specific posttranscriptional microRNAs from the 14q32 locus, which are described to partly affect arteriogenesis and angiogenesis, an effect for CIRP in these processes was proposed [71,72]. However, further in vivo investigations ascertaining that the absence of CIRP actually influences arteriogenesis still need to be conducted.

It has been described that increased tissue damage reflects an increased ischemic signal and is expected to be associated with increased angiogenesis [81]. However, our immunohistological analysis showed a significant increase in endothelial cell proliferation and elevated capillaries to muscle fiber ratio in CIRP -/- mice. So, despite the reduced damaged muscle area, and therefore an even smaller expected ischemic stimulus compared to wildtype controls, the CIRP -/- mice showed increased angiogenesis. Consequently, for the first time, we could show ameliorated angiogenesis in CIRP -/- mice through the exact quantification of immunohistological images and thus approve previous assumptions that CIRP affects the processes of angiogenesis [72,74].

The findings of improved angiogenesis were accompanied by a significant reduction of leukocyte accumulation in ischemic tissue of CIRP -/- mice. Leukocytes infiltrate damaged ischemic tissue to remove ischemia-caused cell debris and promote local inflammation leading to further immune cell recruitment. It is well known that emigrated leukocytes, particularly neutrophils and macrophages, play a significant role in distributing pro-angiogenic factors, including VEGFA, and change the microenvironment through their release of proteases [82,83,84,85].

The reduced amount of leukocytes found in the ischemic tissue of CIRP-knockout mice might be based on the absence of extracellular CIRP. The role of eCIRP as a DAMP is described to promote inflammation by binding to the Toll-like receptor 4 (TLR4)-myeloid differentiation factor 2 (MD2) complex as well as to the triggering receptor expressed on myeloid cells 1 (TREM-1). Both can be found on macrophages and neutrophils. The binding of eCIRP activates macrophages and neutrophils and facilitates the secretion of pro-inflammatory acting cytokines and chemokines [66,86,87]. Furthermore, it was described that eCIRP increases vascular permeability and endothelial cell activation in mice lungs [88]. CIRP gets released into the extracellular compartment by active lysosomal secretion due to hypothermia, hypoxia, and inflammation from various cells [65,66]. Referring to our murine hindlimb ischemia model, we assume that eCIRP is upregulated and released in the ischemic muscle to act as a DAMP, similarly as previously described for other hypoxic and mildly hypothermic conditions [48,65,75,89]. Thus, the absence of eCIRP could result in reduced leukocyte recruitment due to the lack of the pro-inflammatory properties of CIRP and a less leaky endothelial barrier. By investigating sepsis and hemorrhagic shock, the lack of CIRP was shown to be associated with reduced inflammatory response and better healing processes in these disease patterns [49,64,65,66]. These observations match our findings of reduced leukocyte accumulation in ischemic muscle tissue in the absence of CIRP.

It is worth mentioning that not all leukocytes have pro-angiogenic effects on ischemia-induced angiogenesis, and a prolonged infiltration in the inflammatory sites can cause aggravated healing and thus obstruct ischemic tissue restitution [82,83,90]. An accelerated inflammatory phase in wound healing in CIRP deficient mice has been reported [74]. These changes were attributed to TNF-α dynamics, showing an earlier rise and equally earlier fall of its expression in CIRP-knockout mice. Wildtype control showed a prolonged infiltration of leukocytes as well as fewer CD31^+^ cells [74]. Our study may reflect a similar effect of an accelerated inflammation and faster remodeling in ischemia-induced angiogenesis, although further investigations in particular on the kinetics of TNF-α are necessary.

Macrophages are a very plastic cell population; their polarization is not static but changes with different cues of the predominant local environment. In this context, it is important to mention that the M1- and M2-like polarized phenotypes only reflect extremes in the wide range of possible polarizations [91]. The presence of macrophages at the side of ischemic tissue is crucial in supporting angiogenesis [18,92,93,94]. In contrast to the reduced total number of leukocytes (CD45^+^ cells), we did not find any significant difference regarding the number of macrophages (CD68^+^ cells) present in the ischemic tissue of CIRP -/- and wildtype control mice at day 7 after FAL.

At the beginning of the inflammatory phase, infiltrative M0-like polarized monocytes mature to classically activated pro-inflammatory M1-like (CD68^+^MRC1^-^) polarized macrophages responsible for phagocytosis and further leukocyte recruitment. During sprouting angiogenesis, it has been described that macrophages expressing an M1-like phenotype assemble near endothelial tip cells and help to guide the newly formed vessel sprouts [95]. Even though M1-like polarized macrophages express a high amount of VEGFA and TNF-α, two major angiogenic factors, they only show a limited effect on angiogenesis. For cancerous tissue, a role apart from only inducing angiogenesis has been reported for VEGFA. It was described that VEGFA mediated signaling influenced the function of immune cells in cancer [96]. A similar effect could be assumed for VEGFA released by M1-like polarized macrophages in ischemic tissue. Furthermore, the M1-like polarized macrophages cannot induce angiogenesis due to a complexation of the angiogenesis inducing zymogen of matrix metalloproteinase 9 (proMMP-9) by tissue inhibitor of metalloproteinase 1 (TIMP-1) [97]. Nevertheless, pro- as well as anti-angiogenic effects for M1-like polarized macrophages are discussed.

Afterwards, M1-like polarized macrophages repolarize to an alternatively activated regenerative anti-inflammatory M2-like (CD68^+^MRC1^+^) phenotype, which announces the start of the subsequent tissue restoration phase [98,99,100]. M2-like polarized macrophages are essential for the resolution of inflammation and tissue repair [82,101]. In contrast to M1-like polarized phenotype, in M2-like polarized macrophages TIMP-1 is downregulated, allowing the release of potent proMMP-9 without blockage. In this way, they promote tissue remodeling, growth, and angiogenesis [97,102,103,104]. Interestingly, it was described that the inhibition of eCIRP decreased the expression of MMP-9 in macrophages, thereby probably affecting angiogenesis [105]. Our immunohistological analysis evidenced a substantial increase in the percentage of regenerative M2-like polarized macrophages in CIRP-knockout mice compared to CIRP-wildtype mice, whereas the number of inflammatory M1-like polarized macrophages decreased compared to control mice. The increased number of M2-like polarized macrophages found in CIRP deficient mice may indicate that these mice—in contrast to wildtype control mice, which still display a high number of M1-like polarized macrophages - have already moved from the initial inflammatory phase towards the tissue regenerating phase, showing reduced tissue damage. A detailed investigation on the progression of tissue damage, angiogenesis, and the predominant leukocyte accumulation by sacrificing mice at different timepoints over a period of 7 days is necessary to evaluate the hypothesis of an accelerated regeneration in CIRP-deficient mice. This analysis could also resolve the question whether milder inflammatory conditions are responsible for increased angiogenesis or whether the lower inflammation is a direct consequence of improved angiogenesis itself in CIRP-knockout mice.

eCIRP exerts its function predominantly as a DAMP promoting inflammation through the activation of nuclear factor ‘kappa-light-chain-enhancer’ of activated B-cells (NF-κB) and the tyrosine kinase Syk via its binding to TLR4 and TREM-1 [66,87]. Thus, we propose that missing eCIRP in the knockout model lacks the release of pro-inflammatory messengers to support classical M1-like activation in macrophages in the present ischemia model. However, further investigations are necessary. Recent studies have shown that pretreatment of a macrophage cell line with recombinant murine CIRP (rmCIRP) pushed them towards an M2-like phenotype in a dose-dependent manner [106]. However, our own in vitro results showed that application of rmCIRP caused an upregulation of markers indicative for M1-like polarized macrophages and had no effect on the markers indicating M2-like phenotype polarization. Although still unclear, these contradictory findings might be based on the different macrophage cell lines or rmCIRP used in the individual approaches.

In tissue damage, neutrophils belong to the first recruited cells of the innate immune system [84]. They play a pivotal role in the initiation of angiogenesis through their direct allocation of VEGFA and by providing MMPs, especially active MMP-9, leading to the deconstruction of the extracellular matrix and the accompanying release of former matrix-bound VEGFA and further growth factors [32,107,108,109]. Moreover, it was found that neutrophils can promote angiogenesis via the creation of tunnels for new vessel sprouts in a model of sterile thermal hepatic injury [90]. As with macrophages, they are capable of phagocytizing cell debris and regulating inflammation by providing a wide range of pro-inflammatory cytokines and chemokines [110]. For a long time, it was assumed that infiltrated neutrophils are the major cause of aggravated muscle injury [111]. Albeit, it was shown that the depletion of neutrophils in muscle injury interfered with tissue repairing mechanisms, suggesting that neutrophil-dependent damage at the injury site could be essential for tissue restoration [112,113]. After orchestrating the inflammation, removing cell debris, and initiating angiogenesis, neutrophils are not cleared through phagocytosis but have been demonstrated to leave the inflammation site via a process referred to as reverse transendothelial migration, thus re-entering the circulation [114].

Neutrophils are important players in the process of angiogenesis. In our study, however, we found a reduced number of neutrophils in ischemic tissue of CIRP deficient mice along with increased angiogenesis and reduced tissue damage. According to the statements above, the reduced accumulation of neutrophils in CIRP deficient mice might not be due to reduced recruitment of neutrophils but could be the result of an enhanced reverse transendothelial migration. Thus, after initiating angiogenesis and clearing cell debris, neutrophils may have already migrated back into the vasculature, resulting in a reduced number of neutrophils found at the inflammatory site in CIRP deficient mice. Yet, it has been described that eCIRP can induce reverse transendothelial migration in sepsis [115]. Here it is important to mention that in contrast to sterile inflammation, in sepsis reverse transendothelial migration does not lead to tissue restoration but instead promotes the dissemination of a local to systemic inflammation [115]. The assumption that the absence of CIRP fosters transendothelial migration of neutrophils in ischemia-induced tissue damage is an interesting aspect that deserves further investigation.

Our study evidenced a decreased number of NETs and NET-forming neutrophils in the ischemic tissue of CIRP -/- mice compared to wildtype control mice. This is in line with previous findings stating that eCIRP induces NET formation [68,69,70]. Whether pro-inflammatory ICAM-1^+^ neutrophils, which are believed to be induced by eCIRP, are the predominant subpopulation in CIRP-wildtype mice experiencing ischemia and ICAM-1^+^ originated NETs also have the same effect on angiogenesis needs to be further investigated. Since fewer neutrophils were found in the tissue of our knockout model, it is likely to assume that less ICAM-1 positivity resulted in fewer NETs. In our study, we could not prove previous findings showing that increased NET formation results in more angiogenesis [40]. In this context, it is also important to mention that in wound healing, angiogenesis is not associated with NET formation and that NETs can even cause tissue damage [42,43,44,116]. For diabetes, it has been suggested that NET formation may be causative for delayed wound healing [43]. So, from the results of our study, we suggest that CIRP deficiency provokes a milder and more pro-angiogenic and tissue remodeling orientated NET formation and thus improves angiogenesis and tissue restoration.

Interestingly, a recently published study on sepsis showed that CIRP-induced NET-formation declines efferocytosis, i.e., the process of phagocytosis of apoptotic cells by macrophages [117]. This could be the missing link to our results on the overall prolonged inflammatory phase in wildtype mice compared to the presumably accelerated inflammatory phase in CIRP-knockout mice resulting earlier in the M2-like polarized macrophages dominated remolding phase. Increased NET formation would thus not improve angiogenesis but prolong the inflammatory phase by promoting the recruitment of further leukocytes to compensate for the consequences of reduced efferocytosis. Whether the effect of CIRP on efferocytosis observed in sepsis also applies for sterile and ischemic induced tissue damage remains to be investigated and needs to be a topic of further in-depth studies.

Angiogenesis is not only regulated by paracrine factors supplied by leukocytes, but when investigating angiogenesis intracellular effectors must be considered as well. Previous in vitro studies have shown that CIRP can bind to pre-miR-329, suggesting that CIRP plays a role in posttranscriptional regulation of a microRNA relevant for neovascularization [72]. miR-329 is known to downregulate CD146 levels on endothelial cells. CD146 itself is a pro-angiogenic effector and an important VEGFR-2 co-receptor in angiogenesis [71,73]. Moreover, it has been demonstrated that inhibition of miR-329 resulted in increased angiogenesis and arteriogenesis [71]. However, miR-329 is not the only microRNA connected with angiogenesis or arteriogenesis, and it will be interesting to investigate whether CIRP regulates the bioavailability of other microRNAs relevant for vascular cell proliferation and the processes of vascularization as such as well.

How far the observed effects in the present study on angiogenesis as well as on inflammation and tissue regeneration are due to the absence of intracellular or extracellular CIRP remains to be addressed. Administering recombinant CIRP to CIRP deficient mice or blocking studies employing CIRP-specific antibodies in wildtype mice may help to attribute iCIRP and eCIRP defined roles in angiogenesis in tissue regeneration. The results of our study mainly suggest a role for eCIRP regarding leukocyte recruitment, angiogenesis, and tissue remodeling, although it is likely that the absence of iCIRP contributed substantially too.

In summary, we show that the absence of CIRP enhanced the processes of angiogenesis and tissue regeneration. The predominant presence of regenerative M2-like polarized macrophages, the reduced numbers of pro-inflammatory M1-like polarized macrophages, neutrophils, and NETs at the site of ischemia-induced inflammation are strong indicators of milder inflammatory conditions, leading to effective angiogenesis and hence enhanced tissue repair in CIRP deficient mice. We suggest that the absence of eCIRPs role as a DAMP signal influences the observed changes in leukocyte recruitment and macrophage polarization. However, also improved angiogenesis might be causative for accelerated resolution of inflammation. Moreover, further investigations are necessary to ascertain whether improved angiogenesis and tissue remodeling found in CIRP-knockout mice can be attributed to the deficiency of its intracellular function as an RNA-chaperone or to its extracellular functions, which has started to be analyzed in detail recently.

## Data Availability

The data presented in this study is available on request from the first author.

## Supplementary Materials

The following are available online at https://www.mdpi.com/article/10.3390/biomedicines9040395/s1, Figure S1: CIRP mRNA increases significantly 72 h after FAL under conditions of ischemia in gastrocnemius muscle. Figure S2: Tissue samples from sham operated CIRP-knockout and -wildtype mice show no difference in capillary density. Figure S3: CIRP induces the expression of M1-like polarization markers in macrophages but does not influence M2-like polarization markers. J774A.1 macrophages were treated with murine recombinant CIRP at indicated concentrations.
