# Supplementary material for: Absence of Cold-Inducible RNA-Binding Protein (CIRP) Promotes Angiogenesis and Regeneration of Ischemic Tissue by Inducing M2-Like Macrophage Polarization
Source: Biomedicines. 2021 Apr 7;9(4):395. doi: 10.3390/biomedicines9040395 (PMC8067566; doi:10.3390/biomedicines9040395)
Supplement: Supplementary file 1 [file biomedicines-09-00395-s001.pdf]

biomedicines

Article

Absence of Cold-inducible RNA-binding protein (CIRP) promotes angiogenesis and regeneration of ischemic tissue by inducing M2-like macrophage polarization

Matthias Kübler[1,2], Sebastian Beck[1,2], Silvia Fischer[3], Philipp Götz[1,2], Konda Kumaraswami[1,2], Hellen Ishikawa-Ankerhold[1,4,‡], Manuel Lasch[1,2,5,‡], and Elisabeth Deindl[1,2,‡,*]

1 Walter-Brendel-Centre of Experimental Medicine, University Hospital, Ludwig-Maximilians-Universität München, 81377 Munich, Germany; Matthias.Kuebler@med.uni-muenchen.de (M.K.); s.beck1@campus.lmu.de (S.B.); P.Goetz@med.uni-muenchen.de (P.G.); Kumaraswami.Konda@med.uni-muenchen.de (K.K.); Hellen.Ishikawa-Ankerhold@med.uni-muenchen.de (H.I.-A.); manuel_lasch@gmx.de (M.L.); Elisabeth.Deindl@med.uni-muenchen.de (E.D.)
2 Biomedical Center, Institute of Cardiovascular Physiology and Pathophysiology, Faculty of Medicine, Ludwig-Maximilians-Universität München, 82152 Planegg-Martinsried, Germany
3 Department of Biochemistry, Faculty of Medicine, Justus Liebig University, 35392 Giessen, Germany; Silvia.Fischer@biochemie.med.uni-giessen.de (S.F.)
4 Department of Internal Medicine I, Faculty of Medicine, University Hospital, Ludwig-Maximilians-Universität München, 81377 Munich, Germany
5 Department of Otorhinolaryngology, Head and Neck Surgery, University Hospital, Ludwig-Maximilians-Universität München, 81377 Munich, Germany
* Correspondence: Elisabeth.Deindl@med.uni-muenchen.de; Tel.: +49-(0)-89-2180-76504
‡ These authors contributed equally to this work.

Citation: Kübler, M.; Beck, S.; Fischer, S.; Götz, P.; Kumaraswami, K.; Ishikawa-Ankerhold, H.; Lasch, M.; Deindl, E. Absence of Cold-Inducible RNA-Binding Protein (CIRP) Promotes Angiogenesis and Regeneration of Ischemic Tissue by Inducing M2-Like Macrophage Polarization. *Biomedicines* **2021**, *9*, 395. https://doi.org/10.3390/biomedicines9040395

Academic Editor: Alexei Gratchev

Received: 28 February 2021
Accepted: 4 April 2021
Published: 7 April 2021

Publisher's Note: MDPI stays neutral with regard to jurisdictional claims in published maps and institutional affiliations.

Copyright: © 2021 by the authors. Submitted for possible open access publication under the terms and conditions of the Creative Commons Attribution (CC BY) license (http://creativecommons.org/licenses/by/4.0/).

Supplement

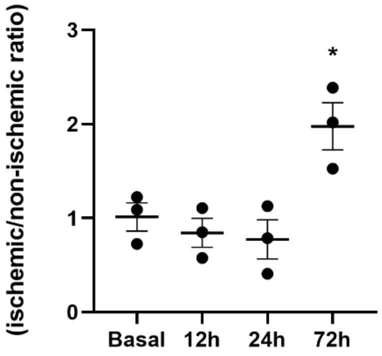

Supplement Figure S1. Cold-inducible RNA-binding protein (CIRP) mRNA increases significantly 72h after femoral artery ligation (FAL) under conditions of ischemia in gastrocnemius muscle. The scatter plot displays the mRNA levels of CIRP in gastrocnemius muscle without ligation (basal), and 12 h, 24 h and 72 h after FAL in relation to sham operation. Results were normalized to the expression level of 18S rRNA. Data are means ± S.E.M., n = 3 in triplicates, * $p < 0.05$ (72h vs. basal) determined by one-way ANOVA with the Bonferroni multiple comparisons test.

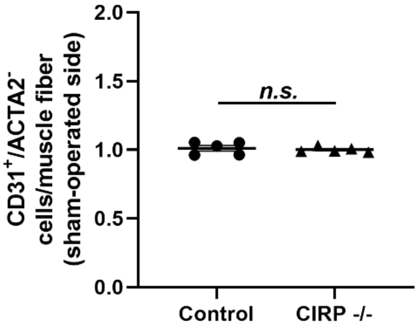

Supplement Figure S2. Tissue samples from sham operated Cold-inducible RNA-binding protein (CIRP) -knockout and -wildtype mice show no difference in capillary density. The scatter plot displays CD31$^+$/ACTA2$^-$ (actin alpha 2) cells per muscle fiber of gastrocnemius muscles from sham-operated sides of CIRP-knockout and wildtype control mice 7 days after femoral artery ligation (FAL). Data are means ± S.E.M., n = 5 per group, n.s. $p > 0.05$ (CIRP -/- vs. control) by unpaired, two-sided student's t-test.

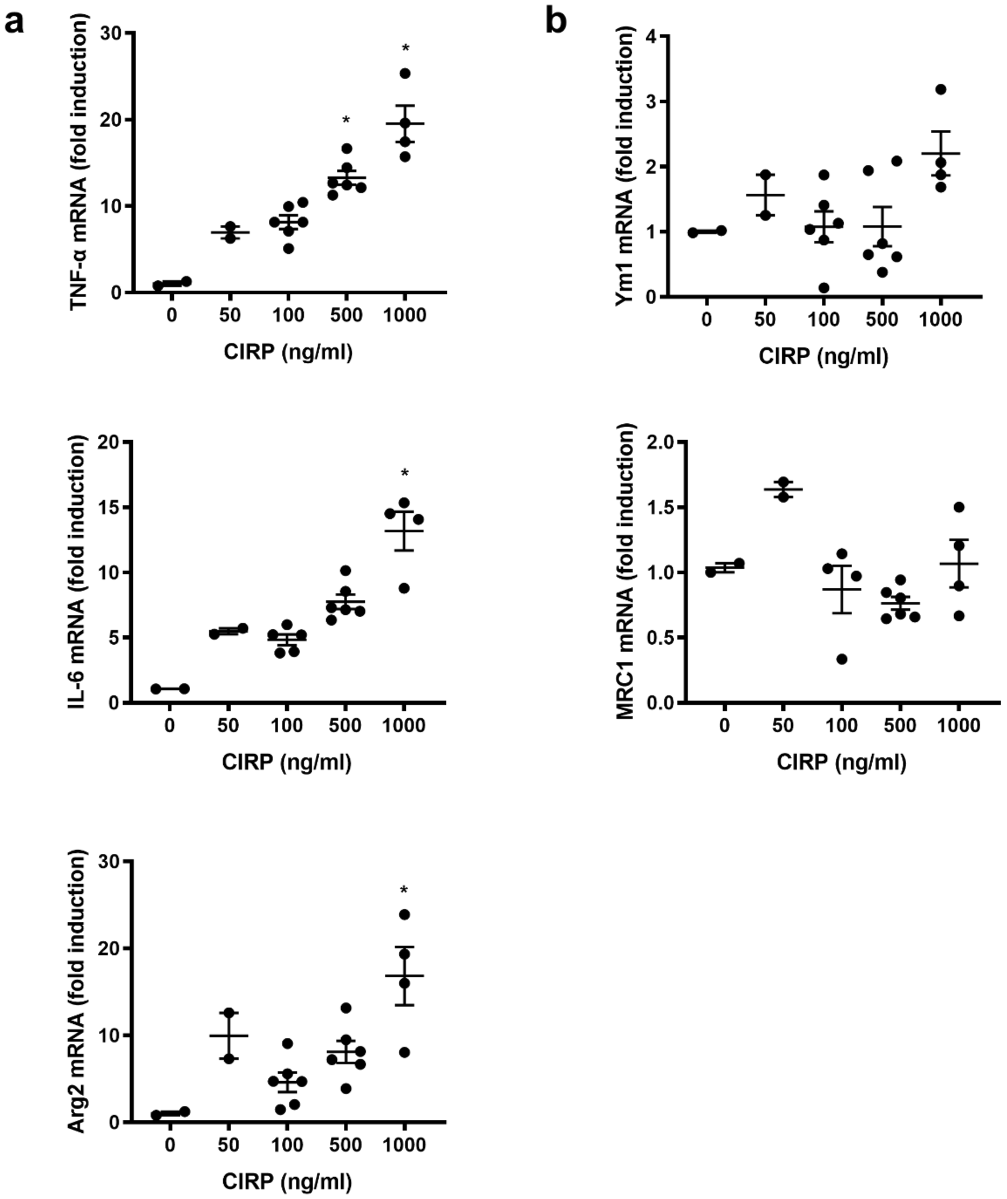

Supplement Figure S3. Cold-inducible RNA-binding protein (CIRP) induces the expression of M1-like polarization markers in macrophages but does not influence M2-like polarization markers. J774A.1 macrophages were treated with murine recombinant CIRP at indicated concentrations. Scatter plots show the results of qRT-PCR analyses on the expression level of **(a)** the M1-like polarization markers: TNF-α (tumor necrosis factor alpha), IL-6 (interleukin 6) and Arg2 (arginase 2), and **(b)** the M2-like polarization markers Ym1 (chitinase-like protein 3) and MRC-1 (mannose receptor C-type 1). Results were normalized to the expression level of actin. Data are means ± S.E.M., n ≥ 2 in duplicates, * $p < 0.05$ compared to 0 ng/ml CIRP determined by 2way ANOVA with the Tukey's multiple-comparisons test.
